# Novel murine models for studying Cache Valley virus pathogenesis and *in utero* transmission

**DOI:** 10.1080/22221751.2021.1965497

**Published:** 2021-08-18

**Authors:** Krisangel López, Sarah N. Wilson, Sheryl Coutermash-Ott, Manette Tanelus, William B. Stone, Danielle L. Porier, Dawn I. Auguste, John A. Muller, Orchid M. Allicock, Sally L. Paulson, Jesse H. Erasmus, Albert J. Auguste

**Affiliations:** a Department of Entomology, College of Agriculture and Life Sciences, Fralin Life Science Institute, Virginia Polytechnic Institute and State University, Blacksburg, VA, USA; b Department of Biomedical Sciences and Pathobiology, Virginia Tech, VA-MD College of Veterinary Medicine, Blacksburg, VA, USA; c Department of Biology, University of Oklahoma, Norman, OK, USA; d Department of Epidemiology of Microbial Diseases, Yale School of Public Health, New Haven, CT, USA; e HDT Bio, Seattle, WA, USA; f Center for Emerging, Zoonotic, and Arthropod-borne Pathogens, Virginia Polytechnic Institute and State University, Blacksburg, VA, USA

**Keywords:** Cache Valley virus, pathogenesis, animal models, murine model, orthobunyavirus

## Abstract

Cache Valley virus (CVV) is a prevalent emerging pathogen of significant importance to agricultural and human health in North America. Emergence in livestock can result in substantial agroeconomic losses resulting from the severe embryonic lethality associated with infection during pregnancy. Although CVV pathogenesis has been well described in ruminants, small animal models are still unavailable, which limits our ability to study its pathogenesis and perform preclinical testing of therapeutics. Herein, we explored CVV pathogenesis, tissue tropism, and disease outcomes in a variety of murine models, including immune -competent and -compromised animals. Our results show that development of CVV disease in mice is dependent on innate immune responses, and type I interferon signalling is essential for preventing infection in mice. IFN-αβR^-/-^ mice infected with CVV present with significant disease and lethal infections, with minimal differences in age-dependent pathogenesis, suggesting this model is appropriate for pathogenesis-related, and short- and long-term therapeutic studies. We also developed a novel CVV *in utero* transmission model that showed high rates of transmission, spontaneous abortions, and congenital malformations during infection. CVV infection presents a wide tissue tropism, with significant amplification in liver, spleen, and placenta tissues. Immune-competent mice are generally resistant to infection, and only show disease in an age dependent manner. Given the high seropositivity rates in regions of North America, and the continuing geographic expansion of competent mosquito vectors, the risk of epidemic and epizootic emergence of CVV is high, and interventions are needed for this important pathogen.

## Introduction

Cache Valley virus (CVV) is an emerging zoonotic mosquito-borne virus, and a member of the Bunyamwera serogroup along with other veterinary and public health important viruses within the genus *Orthobunyavirus* [1–3]. Since CVV's first isolation in 1956, in Cache Valley, Utah [4] it has become an important pathogen in Canada, the U.S., and Mexico, where its emergence has resulted in significant agroeconomic losses. Although CVV emergence has historically been sporadic, it remains an important public health and agricultural concern as exemplified by its recent outbreaks in Canada in 2016 and New York in 2021 [5,6].

CVV virions are enveloped, pleomorphic, and ∼80–120 nm in diameter. Virions encapsidate a tri-segmented, negative sense genome that includes the L segment which encodes a viral RNA polymerase, an M segment that encodes the glycoproteins Gn and Gc and a nonstructural protein (NSm), and an S segment that encodes the nucleocapsid and a small nonstructural protein (NSs) [7]. Currently, there are two phylogenetic lineages of CVV, an ancestral lineage I (1952–2011), and the most recent lineage II (2011–2014) which is thought to have emerged from a Mexican strain isolated in the Yucatan peninsula [8]. Since the discovery of the new lineage II strain, subsequent surveillance studies demonstrate nearly all samples collected in Connecticut in 2014 (i.e. four years after it was identified) are lineage II strains, suggesting a potential replacement event [8].

CVV has an extraordinarily wide host range and has previously been serologically detected or isolated from several domestic animals including sheep (*Ovis aries)*, goats (*Capra hircus*), cattle (*Bos taurus)*, horses (*Equus caballus*), swine (*Sus scrofus*), rabbits (family Leporidae), and guinea pigs (*Cavia porcellus*) [7,9]. There is strong evidence that CVV transmission can be maintained in horses (*Equus caballus*) [10], swine, goats, [11], elk (*Cervus canadensis*) [12], and white-tailed deer (*Odocoileus virginianus*) which are suspected to be the primary amplification host [13,14]. Serological surveys conducted on sheep throughout the U.S. have demonstrated high seropositivity rates for CVV. Rates were as high as 96.7% in the eastern U.S., to 53.3% in the central U.S., and 58.9% in the Western U.S. [15]. CVV infections have been reported in humans [9,16–19] and seroprevalence rates in humans have been shown to be as high as 40% [9,19–22]. The virus is known to cause disease in humans, including fever, headaches, nausea, fatigue, encephalitis, meningitis, spontaneous abortions, and macrocephaly in infants [17–20,23,24]. A unique and critical phenotype underlying the importance of CVV is its teratogenicity. CVV infection during pregnancy causes a range of congenital birth defects in small ruminants, including reproductive failure and/or major congenital defects of the skeletal and central nervous systems [9,11,24–26].

CVV demonstrates a rare vector transmissibility phenotype, and is maintained through horizontal transmission, with some evidence of vertical transmission in multiple mosquito genera, including *Aedes, Anopheles, Culiseta,* and *Coquillettidia* species [27–29]. This has enormous ramifications since these vectors opportunistically feed on most livestock, and/or humans in their proximity. The close association of these vectors with current agricultural practices combined with CVV's high prevalence in nature, suggest a high risk of infection and epizootic/epidemic potential in North America [5].

CVV is an important pathogen and intervention strategies are urgently needed to prevent its emergence. Despite its significance, there are very limited resources and currently no available murine models for studying CVV pathogenesis or evaluating countermeasures. Here, we explored CVV pathogenesis, tissue tropism and disease outcomes in a variety of murine models, including immune -competent and -compromised animals. We also compare the pathogenesis among contemporaneous isolates of both CVV lineages to identify which strain should be preferentially selected as a challenge model for countermeasure testing. Our studies show that development of CVV disease in mice is dependent on innate immune responses and type I interferon signalling is essential for preventing infection in mice. Among the models studied, we also develop a CVV *in utero* transmission model that shows high rates of transmission and spontaneous abortions during infection. Altogether, our novel murine models demonstrate that CVV pathogenesis is type I interferon-dependent, as well as age-dependent in immune-competent mice.

## Materials and methods

### Viruses and cell culture

CVV strains from both genetic lineages were obtained from Dr. Sally Paulson and Dr. Philip Armstrong. Viruses were selected to represent CVV's genetic diversity, ensure they were isolated from different enzootic sources, and have minimal passage histories to reduce cell culture adaptive effects (Table S1). Viruses were propagated in Vero-76 (African Green Monkey Kidney) and C6/36 (*Ae. albopictus*) cells (ATCC; Manassas, VA, USA) and titers are shown in Table S1. Briefly, 80% confluent cell monolayers were inoculated with an aliquot of each virus strain, and cultures were harvested when 50% cytopathic effects (CPE) were observed in Vero-76 cells. Culture supernatants were collected, clarified by centrifugation at 3345 × g for ten minutes at 4°C and stored at −80°C. Virus titers were estimated by plaque assays on Vero-76 cells as previously described [30]. Complete genome sequences for 4B and W08491 were determined using next-generation sequencing as previously described [31], and sequences are available in GenBank under accession numbers MZ612419, MZ612420 and MZ612423 for 4B and MZ612418, MZ612421 and MZ612422 for W08491.

### CVV pathogenesis in immune-compromised IFN-αβR^-/-^ mice

Six-week-old IFN-αβR^-/-^ (interferon alpha and beta receptor 1 knockout on a BL6 background) mice were purchased from The Jackson Laboratory (Bar Harbor, ME, USA; Mouse strain: B6 129S2 -*ifnar1*^tm1Agt^/Mmjax) and a colony maintained at Virginia Tech. Mice were separated into three groups including CVV 4B (*n* = 14), CVV W08491 (*n* = 14), and phosphate buffered saline (PBS) (*n* = 13; unchallenged healthy controls) and inoculated subcutaneously with 10^4^ plaque forming units (PFU) of CVV, or with PBS diluent. Mice were inoculated subcutaneously as previously done with ruminants and to reflect a natural peripheral infection route [32]. Mice were bled retro-orbitally for the first four days post infection (DPI) to assess viremia. Blood samples were separated by centrifugation at 3099 × g for five minutes, and serum samples were labelled and stored at −80°C until plaque assays were performed. After challenge, mice were monitored for weight change, signs of disease, and mortality for 14 days. Necropsies were conducted on 3 mice per group on 3 and 5 DPI. Tissues were collected, half of which were fixed in 10% formalin solution for histological analysis, and the other half stored in culture media (i.e. Dulbecco's Modified Eagle Media (DMEM) containing 2% Fetal Bovine Serum (FBS), 100 units of Penicillin and 0.1 mg Streptomycin), for virus quantification by plaque assay. Mice were immediately euthanized when moribund or upon weight loss greater than or equal to 20% of their original body weight, as previously described [33].

Three-week old mice were separated into three groups CVV 4B (*n* = 9), CVV W08491 (*n* = 9), and PBS (*n* = 9; unchallenged healthy controls). Mice were inoculated, bled, monitored, and euthanized as above except that this study lasted 10 days. Necropsies and tissue collections were done on 3 DPI, and tissues processed as above.

One-year old mice were separated into three groups CVV 4B (*n* = 12), CVV W08491 (*n* = 12) and PBS (*n* = 8; unchallenged healthy controls). Mice were challenged with 10^4^ PFU of CVV or PBS diluent. Mice were bled retro-orbitally on 2, 3, 4 and 5 DPI to assay for viremia. After challenge, weight change, mortality and signs of disease were monitored for 6 days. Necropsies were performed on 2, 4, and 6 DPI, with half of the tissues fixed in 10% formalin solution for histological analysis, and the other half stored in media for virus quantification as above.

### In utero transmission of CVV in IFN-αβR^-/-^ mice

Mice were separated into three groups CVV 4B (*n* = 8, CVV W08491 (*n* = 9) and PBS (*n* = 7). Adult IFN-αβR^-/-^ dams were mated and monitored daily for vaginal plugs. Upon detection of vaginal plugs, embryonic development day E0.5 was noted. Mice were challenged subcutaneously ten days later (E10.5) with 10^4^ PFU of CVV or PBS diluent. Dams were monitored and weighed daily and retro-orbitally bled on 4 DPI, followed by euthanasia and necropsies on 5 DPI. Maternal tissues (brain, liver, spleen, placenta, kidney, heart, and lung) and fetus heads were harvested from each dam, weighed, and stored in media until assayed for virus. Tissues were assayed for virus as described above.

### Histopathology and organ loads in harvested tissues

Harvested tissues were homogenized using a Qiagen TissueLyser (Qiagen, Germantown, MD) for 5 min at 26,000 Hz and clarified by centrifugation for 5 min at 5510 × g. Samples were then titrated by plaque assays on Vero-76 cells to estimate organ virus loads as above. Virus groups were blinded, tissues were formalin fixed, paraffin embedded, sectioned, and hematoxylin and eosin (H&E) stained by the ViTALS diagnostic lab (Virginia Tech, Blacksburg, VA, USA). Pathology was scored blind by a board-certified veterinary pathologist. All tissues were graded individually for the presence and degree of cellular degeneration, cell death, and inflammation. Each was graded on a scale from 0 to 3 where 0 = no lesions observed, 1 = mild lesions observed, 2 = moderate lesions observed, and 3 = severe lesions observed. Individual scores for each parameter were then summed for each tissue for a total histopathology score for each organ.

### Hemogram sysmex and Immunoassay in IFN-αβR^-/-^ mice

Three-month old mice were separated into three groups, including CVV 4B (*n* = 15), CVV W08491 (*n* = 15), and PBS (*n* = 15). Mice were challenged with 10^4^ PFU of CVV or PBS diluent. On 1, 3, and 5 DPI, five mice from each group were euthanized and cardiac bleeds performed. Whole blood (300 μl) was placed into EDTA microtainer tubes and mixed thoroughly to avoid clotting. Samples were sent to the ViTALS Animal Laboratory (Virginia Tech) and a full hemogram panel performed. Sera (*n* = 12 per group) collected on 1, 3 and 5 DPI were analysed using a FirePlex 96-Key Cytokines Mouse Immunoassay for 17 cytokines/chemokines (Abcam, Cambridge, MA). Samples were run in duplicate at a 1:2 dilution.

### CVV pathogenesis in immune-competent CD-1 suckling mice

A CD-1 suckling mouse model was used to assess CVV neurovirulence, potential differences in strain pathogenicity, and compare the pathogenicity of these strains to strains used in previous studies. Six E17 pregnant dams were purchased from Charles River Laboratories (Strain: CD-1® IGS; Wilmington, MA, USA). Dams were equally divided into three groups CVV 4B (*n* = 2), CVV W08491 (*n* = 2) and PBS (*n* = 2; unchallenged healthy controls) and allowed to acclimatize and birth pups. Two days after birth, pups were intracranially inoculated with ∼10^4^ PFU of respective virus, or PBS diluent. Suckling mice were weighed and monitored daily for 14 DPI for signs of disease. On 3 and 5 DPI, brains were harvested from 3 mice per group for histopathology and virus quantification to assess replication kinetics. Upon visual manifestation of disease (cyanosis, unresponsiveness) or on collection days, mice were euthanized.

### CVV pathogenesis in seven-week-old immune-competent C57BL/6J mice

C57BL/6J mice were obtained from The Jackson Laboratory (Strain: B6; Bar Harbor, ME, USA) and separated into five groups CVV 4B + MAR1-5A3 (*n* = 8), CVV 4B + Isotype IgG_1_ Control (*n* = 8), CVV W08491+ MAR1-5A3 (*n* = 8) CVV W08491+ Isotype IgG_1_ Control (*n* = 8), and PBS (*n* = 8; unchallenged healthy controls). One day prior to inoculation, mice were administered 2.5 mg of either MAR1-5A3, Isotype IgG_1_ Control (Leinco Technologies; St. Louis, MO, USA) or PBS intraperitoneally. Mice were then subcutaneously inoculated with 10^4^ PFU of CVV or PBS diluent. Following infection, mice were given two additional 1 mg doses of MAR1-5A3, Isotype IgG_1_, or PBS on 1 and 4 DPI. Mice were bled retro-orbitally daily for 4 DPI to assess viremia. After challenge, mice were monitored for weight changes, signs of disease, and mortality for 14 days.

### Statistical analysis

Data normality was assessed using a combination of Q-Q plot and box-plot analyses. One-way ANOVAs and mixed-effects analyses followed by a Dunnett's multiple comparison test, and Logrank Mantel–Cox tests were performed using GraphPad Prism version 9.1.0.

## Results

### CVV infection results in significant morbidity and mortality in immune-compromised IFN-αβR^-/-^ mice

**Figure 1.**
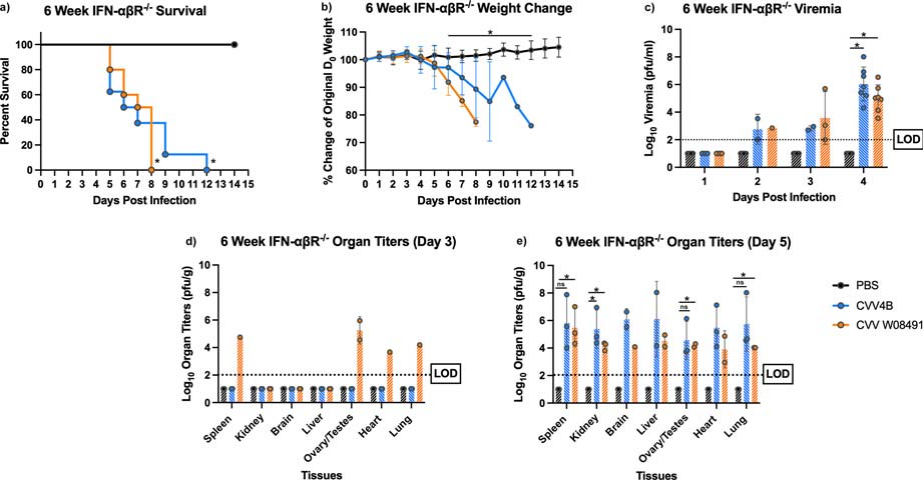
Cache Valley Virus (CVV) infection results in significant viremia, morbidity, and mortality in 6-week-old IFN-αβR^-/-^ mice. Six-week-old mice (CVV 4B *n* = 14, CVV W08491 *n* = 14, PBS *n* = 13) were challenged subcutaneously with 10^4^ plaque forming units (PFU) of virus, and (a) survival and (b) weight change measured daily for 14 days post infection (DPI). (c) Viremia was measured on 1–4 DPI, and organ titers for spleen, kidney, brain, liver, ovary/testes, heart and lung were measured on (d) 3 DPI and (e) 5 DPI. Each data point plotted represents the mean values and error bars indicate standard deviation. The limit of detection (LOD) is indicated with a dotted line. Statistical significance among groups were analysed by log-rank (Mantel-Cox) test in (a), and a mixed effects analysis with a Dunnett's multiple comparison test in (b–e). Statistically significant values are denoted by * (*p* < 0.05).

To explore CVV pathogenesis and potential strain-specific differences in murine pathogenesis, 3- and 6-week-old IFN-αβR^-/-^ mice were subcutaneously inoculated with CVV or PBS diluent. Six-week-old mice began showing signs of illness on 5 DPI and symptoms worsened until euthanasia. Signs of disease included lethargy, disorientation, hunched posture, and sunken eyes, eventually leading to paralysis and unresponsiveness. Mice in the W08491 group succumbed to illness by 8 DPI. However, the 4B group demonstrated 100% mortality by 12 DPI (Figure 1(a)). Mice from both infectious groups showed rapid weight loss starting 5 DPI (Figure 1(b)). Both groups presented significant viremia, achieving titers as high as 8.01 log_10_ PFU/ml for 4B and 6.53 log_10_ PFU/ml for W08491 4 DPI (Figure 1(c)). CVV was detected in all tissues, with the liver and spleens showing the greatest viral loads. Similarly, histopathologic examination of liver and spleen showed that lesions were present in both 3-week and 6-week-old CVV-infected mice. At all time points, histopathologic scores for the spleen and liver of CVV-infected mice were higher than the PBS groups (data not shown).

**Figure 2.**
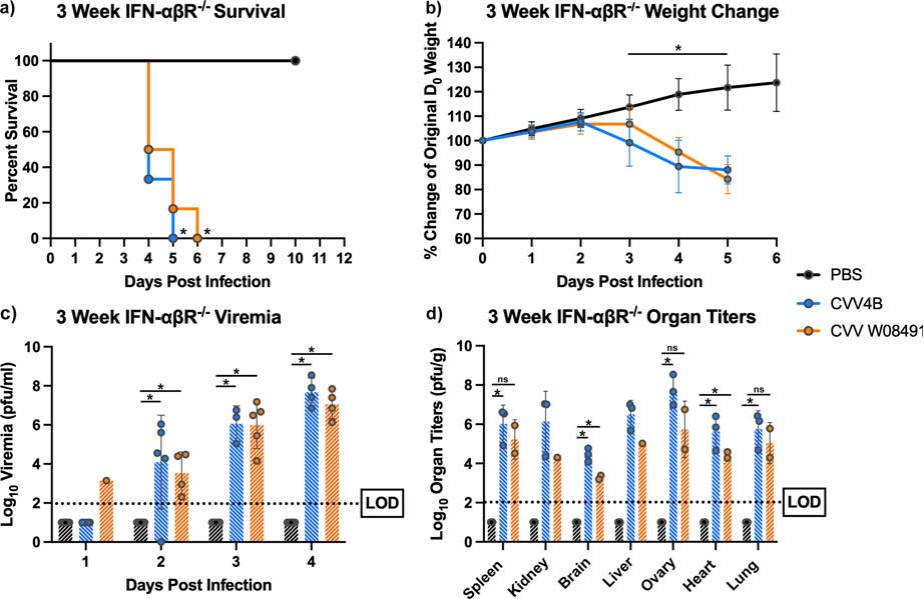
Cache Valley Virus (CVV) infection results in significant morbidity and mortality in 3-week-old IFN-αβR^-/-^ mice. Three-week-old mice (*n* = 9/group) were challenged subcutaneously with 10^4^ plaque forming units (PFU) of virus and (a) survival and (b) weight change measured daily for 10 days post infection (DPI). (c) Viremia was measured on 1–4 d post infection, and (d) organ titers measured for spleen, kidney, brain, liver, ovary, heart and lung harvested 3 DPI. Each data point plotted represents the mean values and error bars indicate standard deviation. The limit of detection (LOD) is indicated with a dotted line. Statistical significance among groups were analysed by log-rank (Mantel-Cox) test in (a), and a mixed effects analysis with a Dunnett's multiple comparison test in (b–d). Statistically significant values are denoted by * (*p* < 0.05).

Both viruses caused significant and rapid mortality in 3-week-old mice, with 100% mortality by 5 DPI in the 4B group, and 6 DPI for the W08491 group (Figure 2(a)). Rapid weight loss was observed in both CVV groups starting on 3 DPI until euthanasia (Figure 2(b)). Viremia was detected at high levels, with both viruses presenting comparable titers on all days except on day 1 post infection, where viremia was only detected in the W08491 group (*n* = 1) (Figure 2(c)). Both virus groups showed high organ titer loads, with spleen, liver and ovaries presenting with the highest viral loads (Figure 2(d)). The 4B group showed the highest organ titers across all tissues (*p* < 0.05). This is supported by the histopathology that showed the highest lesion scores for the liver and spleen were encountered in the 4B groups (data not shown). No significant microscopic lesions were observed in the kidney, lung, or reproductive organs of CVV-infected mice when compared to the PBS group.

### CVV infection and tissue tropism in 1-year-old IFN-αβR^-/-^ mice

**Figure 3.**
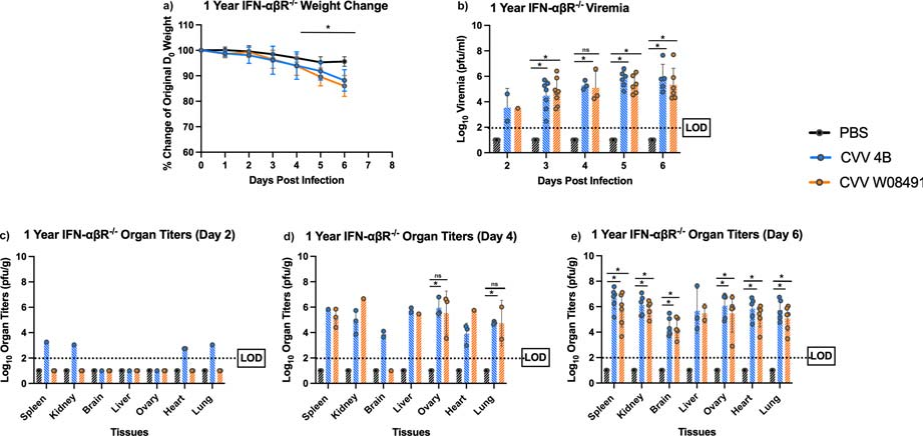
Cache Valley Virus (CVV) infection results in significant viremia, morbidity, and mortality in 1-year-old IFN-αβR^-/-^ mice. One-year-old mice (CVV 4B *n* = 12, CVV W08491 *n* = 12, PBS *n* = 8) were challenged subcutaneously with 10^4^ plaque forming units (PFU) of virus and (a) weight change was measured daily for 6 days post infection (DPI). (b) Viremia was measured on 2–6 DPI, and organ titers for spleen, kidney, brain, liver, ovary, heart and lung at (c) 2 DPI, (d) 4 DPI, and (e) 6 DPI. Each data point plotted represents mean values, and error bars indicate standard deviation. The limit of detection (LOD) is indicated with a dotted line. Statistical significance among groups were analysed by a mixed effects analysis with a Dunnett's multiple comparison test in (a-e). Statistically significant values are denoted by * (*p* < 0.05).

**Figure 4.**
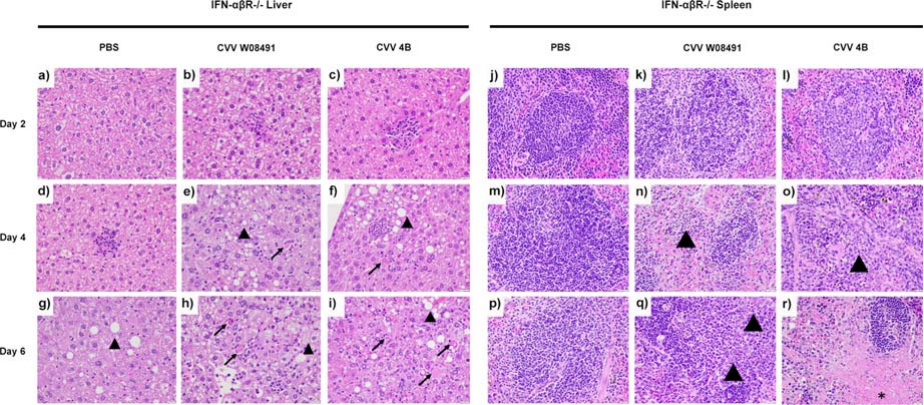
Cache Valley Virus (CVV) infection shows histological effects in livers and spleens of 1-year-old IFN-αβR^-/-^ mice. H&E-stained micrographs of liver (a–i) and spleen (j–r). Liver sections were evaluated for evidence of hepatocyte degeneration characterized by vacuolation of the cytoplasm (arrowhead); cell death characterized by cell swelling, hypereosinophilia of the cytoplasm, and fragmentation of the nucleus (arrow); and degree of inflammation which was predominantly composed of neutrophils and variable numbers of macrophages. Spleen sections were evaluated for inflammation which was predominantly composed of neutrophils (arrowheads) as well as evidence of cell death characterized by necrosis and cellular debris (asterisk). Images were captured at 40x magnification.

To further dissect CVV's tissue tropism and assess age-dependence, we inoculated 1-year old IFN-αβR^-/-^ mice subcutaneously. Weight loss was observed in both CVV groups starting 4 DPI (Figure 3(a)). Viremia was observed in both virus-infected groups on days 2–6 DPI (Figure 3(b)). However, for organ viral loads, only 4B (*n* = 2) showed low titers in various tissues on day 2 post infection (Figure 3(c)), including the spleen, kidney, heart and lungs. By 4 DPI, both 4B and W08491 showed significant virus loads in all tissues with the exception of the brain, where no virus was detected in the W08491 challenged mice (Figure 3(d)). At 6 DPI, all organs demonstrated higher virus titers, with the highest titers observed in the liver, spleen, kidney, and reproductive tissues. The lowest titers were observed in the brain (Figure 3(e)). Similar trends were observed with histological samples. CVV-infected groups began to show lesions in the liver by 2 DPI while lesions in the spleen were not observed until 4 DPI. For both organs, lesions became more severe with time and were the most significant by 6 DPI (Figure 4). Minimal, if any, lesions were observed in the PBS groups. No significant microscopic lesions were observed in the kidney, lung, or reproductive organs of CVV-infected mice when compared to the PBS group.

### In utero CVV transmission causes severe morbidity and mortality in pregnant IFN-αβR^-/-^ dams and fetuses

**Figure 5.**
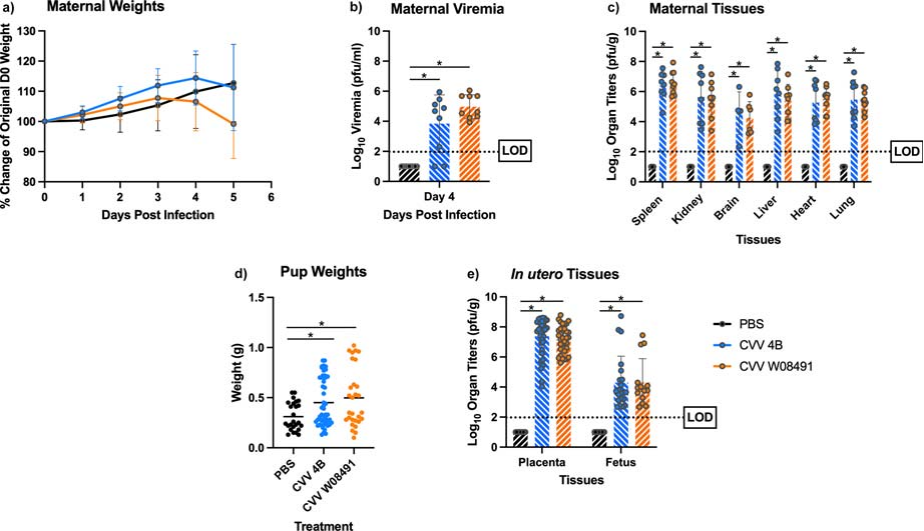
Cache Valley Virus (CVV) infection of IFN-αβR^-/-^ mice results in high in *utero* transmission rates and congenital abnormalities. Pregnant IFN-αβR^-/-^ dams (CVV 4B *n* = 8, CVV W08491 *n* = 9, PBS *n* = 7) were challenged subcutaneously with 10^4^ plaque forming units (PFU) of virus and (a) weight change measured daily for 5 days post infection (DPI). (b) Viremia was measured on 4 DPI. (c) Maternal organ titers for spleen, kidney, brain, liver, ovary, heart and lung, (d) pup weights, and (e) *in utero* tissues, including placenta and fetus brains were assayed for virus titers by plaque assays on Vero-76 cells. Each data point plotted represents the mean values and error bars indicate standard deviation. The limit of detection (LOD) is indicated with a dotted line. Statistical significance among groups were analysed by a mixed effects analysis with a Dunnett's multiple comparison test in (a,c,e), one-way ANOVA with Dunnett's multiple comparison test in (b,d), Statistically significant values are denoted by * (*p* < 0.05).

To further explore CVV's teratogenicity phenotype, we explored *in utero* transmission of CVV and its effect on fetal development. We inoculated pregnant IFN-αβR^-/-^ dams subcutaneously with a respective CVV strain or PBS diluent 10.5 days after the onset of embryonic development (E10.5). Pregnant dams in the W08491 group showed weight loss starting 3, and 4 DPI in the 4B group (Figure 5(a)). Viremia was observed in all CVV-infected groups (Figure 5(b)) confirming infection. High viral loads were detected in all maternal tissues taken at necropsy, 5 DPI (Figure 5(c)). Interestingly, there were statistically significant differences in weights between CVV infected neonates and PBS controls (Figure 5(d)). High viral loads were observed in the placentas of infected dams and associated brains of their neonates. Virus titers were nearly 10,000-fold higher in the placentas when compared to the fetus brains (Figure 5(e)) and 100-fold higher when compared to viremia during necropsy. *In utero* transmission rates to neonates were as high as 60% for W08491- and 46% for 4B-infected mice.

### CVV infection causes thrombocytopenia, lymphocytopenia and dysregulated cytokine responses in IFN-αβR^-/-^ mice

To examine hematological effects of CVV, IFN-αβR^-/-^ mice were challenged with CVV or PBS diluent. CVV 4B infected mice showed statistically significant differences in white blood cells (WBC), eosinophils, lymphocytes and platelets in comparison to PBS controls (Table S2). No differences were observed between the CVV W08491 and the PBS controls. None of the remaining 12 measurements taken showed significant differences between virus infected and PBS control mice. Thrombocytopenia and lymphocytopenia were pronounced at 5 DPI in the W08491 group. CVV 4B and W08491 showed statistically significant differences in CXCL1, GM-CSF, IFN-γ, IL-10, IL-17A, IL-4, IL-5, IL-9, MCP1, MIP1α, and MIP1β when compared to PBS controls (Table S4).

### CVV replicates efficiently and is neurovirulent in immune-competent suckling mice

**Figure 6.**
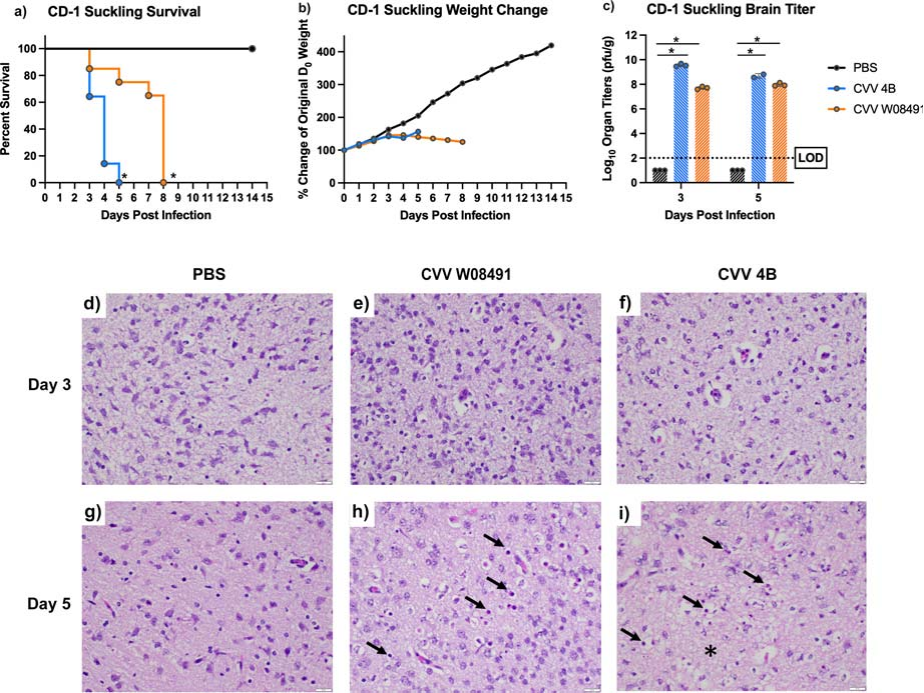
Cache Valley Virus (CVV) infection produces high viral loads and is lethal in CD-1 suckling mice. Two-day-old suckling mice (CVV 4B *n* = 28, CVV W08491 *n* = 23, PBS *n* = 15) were challenged intracranially with 10^4^ plaque forming units (PFU) and (a) survival and (b) weight change measured daily for 14 days post infection (DPI). (c) Virus titers were measured in brain sections harvested on 3 and 5 DPI. Each data point plotted represents the mean values, and error bars indicate standard deviation. The limit of detection (LOD) is indicated with a dotted line. Statistical significance among groups was analysed by logrank (Mantel-Cox) test in (a) a mixed effects analysis with a Dunnett's multiple comparison test in (b,c) Statistical significance is denoted by * (*p* < 0.05). Brain sections were analysed for histopathological effects after H&E staining 3 and 5 DPI. Sections were evaluated for degree of necrosis characterized by neuropil hyalinization and vacuolation (asterisk) as well as shrinkage of cells and condensation of nuclei (arrows). Sections were also evaluated for degree of inflammation as well which was minimal and mostly characterized by rare infiltration by neutrophils (not shown), on (d,e,f) 3 DPI and (g,h,i) 5 DPI. Images were captured at 40x magnification.

We investigated CVV neurovirulence by intracranially inoculating 2-day old CD-1 suckling mice. Mice were inoculated with 10^4^ PFU of CVV 4B (*n* = 28), CVV W08491 (*n* = 23) or PBS (*n* = 15). By 4 DPI, all of the mice in the CVV 4B group had to be euthanized due to severe disease (i.e. cyanotic or unresponsive) (Figure 6(a)). All mice steadily gained weight until 3 DPI (Figure 6(b)), after which CVV 4B-infected mice began to show symptoms of disease and required euthanasia. The CVV W08491 mice began to show symptoms at 7 DPI, and mice were euthanized on 8 DPI due to severe disease (hypoxia, lethargy, moribund). Brain samples harvested from mice (*n* = 3) on 3 and 5 DPI to evaluate organ load titers showed high titers peaking at 9.52 log_10_ PFU/g for CVV 4B and 8.13 log_10_ PFU/g for CVV W08491 infected mice (Figure 6(c)). Histological analysis of brain from CVV-infected mice showed evidence of white matter vacuolation, cell death, and inflammation characterized predominantly by neutrophils (Figure 6(d–i)). Histopathological scores for tissues analysed in all IFN-αβR^-/-^ studies are shown in Table S3.

### CVV infection causes reduced disease in C57Bl/6J mice

To explore CVV pathogenesis in an immune-competent mouse model, we subcutaneously inoculated C57BL/6J mice following an isotype or anti-IFN antibody blockade. Mice were administered MAR1-5A3, Isotype IgG1 control or PBS as described in the methods. All mice survived the 14-day study except for one female in the CVV 4B MAR1-5A3 group which had to be euthanized on 9 DPI. The CVV 4B mice administered MAR1-5A3 showed moderate weight loss (Figure S1), but no disease was observed among any of the virus-infected groups throughout this study. No viremia was detected in this model 1– 4 DPI (data not shown).

## Discussion

CVV is a prevalent emerging pathogen of significant importance to agricultural and human health in North America. Emergence in livestock can result in severe agroeconomic losses due to severe embryonic lethality associated with infection during pregnancy. Although CVV pathogenesis has been well described in ruminants [1,25,32,34], small animal models are still unavailable, which limits our ability to study CVV pathogenesis and perform preclinical testing of therapeutics. Thus, we explored various immune -competent and -compromised murine models using two CVV strains from both phylogenetic lineages.

Herein we report the development of a variety of novel CVV infection and disease murine models. Results from our CD-1 suckling mouse model show comparable mortality to previous studies [4,35]. Previously, CVV was isolated and/or amplified by intracranial inoculation of suckling mice, and mortality was observed in mice 6–13 DPI, although faster mortality rates were observed with mouse-adapted strains. Our model showed exceptional sensitivity and was able to distinguish differences in neurovirulence between both CVV strains studied here. This model should therefore be preferentially employed when comparing neurovirulence among strains. The IFN-αβR^-/-^ models used presented with significant disease and lethal infections, with minimal differences in age-dependent pathogenesis. The IFN-αβR^-/-^ model is an especially susceptible model for arbovirus infections and has been widely employed for pathogenesis and therapeutic testing [36]. This model is therefore appropriate for short- or long-term prophylactic and therapeutic studies. CVV infection of IFN-αβR^-/-^ mice resulted in pronounced thrombocytopenia and lymphopenia, as well as viral infection derived characteristics such as depleted WBCs and nucleated red blood cells (NRBC). NRBCs are generally low in healthy mice but can be increased or severely decreased in pathologic states of any kind. Our progressive timepoints (days 1, 3, 5) showed a decrease which may be associated with the histopathologic inflammation observed. This has previously been recorded in human infections with CVV and other bunyavirus infections [16–19], which provides further support of an accurate reflection of CVV disease in this model. The immunoassays showed the most significant cytokine/chemokine responses in CVV-infected IFN-αβR^-/-^ mice at 3 and 5 DPI. These data suggest that CVV elicits significant pro- and anti -inflammatory cytokine responses, and also strong support for CVV activation of signalling for recruitment of inflammatory cells.

The *in utero* transmission study shows high rates of transmission to neonates. Particularly noteworthy is the fact that IFN-αβR^-/-^ dams experienced spontaneous abortions 5 days after challenge. This abortive effect is a characteristic of CVV in ruminants [1,26,32]. The significant viral titers estimated from placental tissues suggest that CVV is particularly fit for replication in these tissues. This increased replication likely resulted in the large virus burden detected in neonatal brains. CVV infected neonates show statistically significant increased weights in comparison to control neonates; which might suggest congenital abnormalities such as macrocephaly, hydranencephaly, or other developmental defects in infected neonates (Figure 5(d)). Macrocephaly was previously observed in human newborns infected with CVV [23]. These studies confirm observational studies related to CVV outbreaks over the past 40 years, demonstrating severe neurological and physical deformities in ruminant fetuses and humans [21,23,25,32]. Although arthrogryposis (i.e. congenital joint contracture that results in curving of joints) is another common symptom in neonates infected with CVV [26,32,37,38], we did not assess musculoskeletal defects of CVV-infected IFN-αβR^-/-^ neonates. This should be further explored in this model. Further studies exploring the effects of CVV infections at different gestational periods in IFN-αβR^-/-^ mice are still needed, as previously done in sheep [32]. Overall, this model can be a useful tool for understanding the mechanisms underlying the induction of spontaneous abortions, *in utero* transmission and assessing therapeutic efficacy.

C57BL/6J mice are resistant to CVV infection, as seen previously with outbred mice [39,40], likely due to an inability of CVV to antagonize murine innate immune responses. Despite administering a large type I interferon blockade, C57BL/6J mice did not present with viremia or significant mortality, commonly observed with IFN-αβR^-/-^ mice. However, it must be noted that the type I interferon blockade was not evaluated for its efficacy in this study. Weight loss was the only disease signal observed in this model. Further studies are needed to determine if different challenge doses, additional innate immune response knockouts, or different interferon blockade regimens can increase disease in this model. Additionally, future studies using knockout mice such as STAT1^-/-^ and STAT2^-/-^ that are susceptible to orthobunyavirus infection [40] are also needed to identify which innate response pathways restrict CVV replication in mice.

Although CVV replicates efficiently when administered directly to the brain, our data shows lower titers when comparing brain samples to other organs when inoculated peripherally. Our studies show that CVV replicated to the highest titers in spleens, livers, ovaries, and placentas after peripheral inoculation. This suggests CVV has a wide tissue tropism and may be viserotropic. Future studies are needed to determine if different infection routes play a role in CVV tissue tropism.

Recent studies suggest that there has been a lineage replacement event in the northeastern U.S.A., and that lineage II strains now predominate the north eastern U.S. [8]. Despite only using 2 strains, our studies suggest that 4B (lineage II) may be more neurovirulent than W08491 (lineage I). Altogether, our studies show limited differences in disease among strains, with the exception of increased viral loads and neurovirulence with 4B. Further studies that include larger numbers of strains from both phylogenetic lineages are needed to rigorously determine if altered pathogenesis among strains could have influenced the lineage displacement in the northeastern U.S.A.

In conclusion, our results indicate that IFN-αβR^-/-^ mice are a useful tool for studying CVV pathogenesis and *in utero* transmission and can help inform therapeutic and vaccine testing. Immune-competent mice are generally resistant to infection and only show disease in an age dependent manner. Our studies have provided valuable details on important characteristics of CVV infection, pathogenesis, and immune responses in relevant and commercially available mouse models. With seropositivity rates in parts of the eastern USA as high as 96.4% in sheep [5,15], and the continuing geographic expansion of mosquitoes capable of transmitting the pathogen, like *Ae. aegypti* and *Ae. albopictus* [9], the risk of emergence for CVV is high, and interventions are urgently needed.

## Supplementary Material

Supplemental Material

Supplementary_data_set.xlsx

## References

[1] Edwards JF. Cache Valley virus. Vet Clin North Am Food Anim Pract. 1994;10(3):515–524. 7728634 10.1016/s0749-0720(15)30536-3

[2] Calisher CH, Francy DB, Smith GC, et al. Distribution of Bunyamwera serogroup viruses in North America, 1956–1984. Am J Trop Med Hyg. 1986 Mar;35(2):429–443. 2869708 10.4269/ajtmh.1986.35.429

[3] Noronha LE, Wilson WC. Comparison of two zoonotic viruses from the order Bunyavirales. Curr Opin Virol. 2017 Dec;27:36–41. 29128744 10.1016/j.coviro.2017.10.007

[4] Holden P, Hess AD. Cache Valley virus, a previously undescribed mosquito-borne agent. Science. 1959 Oct 30;130(3383):1187–1188. 14402567 10.1126/science.130.3383.1187

[5] Uehlinger FD, Wilkins W, Godson DL, et al. Seroprevalence of Cache Valley virus and related viruses in sheep and other livestock from Saskatchewan, Canada. Can Vet J. 2018 Apr;59(4):413–418. 29606729 PMC5855288

[6] Cornell University CoVM. Cache Valley Virus. 2021.

[7] Brockus CL, Grimstad PR. Sequence analysis of the medium (M) segment of Cache Valley virus,1 with comparison to other Bunyaviridae. Virus Genes. 1999;19(1):73–83. 10499453 10.1023/a:1008144808041

[8] Armstrong PM, Andreadis TG, Anderson JF. Emergence of a new lineage of Cache Valley virus (Bunyaviridae: Orthobunyavirus) in the Northeastern United States. Am J Trop Med Hyg. 2015 Jul;93(1):11–17. 25962774 10.4269/ajtmh.15-0132 PMC4497881

[9] Waddell L, Pachal N, Mascarenhas M, et al. Cache Valley virus: A scoping review of the global evidence. Zoonoses Public Health. 2019 Nov;66(7):739–758. 31254324 10.1111/zph.12621 PMC6851749

[10] McLean RG, Calisher CH, Parham GL. Isolation of Cache Valley virus and detection of antibody for selected arboviruses in Michigan horses in 1980. Am J Vet Res. 1987 Jul;48(7):1039–1041. 3631684

[11] Kokernot RH, Hayes J, Tempelis CH, et al. Arbovirus studies in the Ohio-Mississippi Basin, 1964–1967. IV. Cache Valley virus. Am J Trop Med Hyg. 1969 Sep;18(5):768–773. 5817892 10.4269/ajtmh.1969.18.768

[12] Hoff GL, Spalatin J, Trainer DO, et al. Isolation of a bunyamwera group arbovirus from a naturally infected caribou. J Wildl Dis. 1970 Oct;6(4):483–487. 16512161 10.7589/0090-3558-6.4.483

[13] Blackmore CG, Grimstad PR. Cache Valley and Potosi viruses (Bunyaviridae) in white-tailed deer (Odocoileus virginianus): experimental infections and antibody prevalence in natural populations. Am J Trop Med Hyg. 1998 Nov;59(5):704–709. 9840585 10.4269/ajtmh.1998.59.704

[14] Neitzel DF, Grimstad PR. Serological evidence of California group and Cache Valley virus infection in Minnesota white-tailed deer. J Wildl Dis. 1991 Apr;27(2):230–237. 1906113 10.7589/0090-3558-27.2.230

[15] Meyers MT, Bahnson CS, Hanlon M, et al. Management factors associated with operation-level prevalence of antibodies to Cache Valley virus and other Bunyamwera serogroup viruses in sheep in the United States. Vector Borne Zoonotic Dis. 2015 Nov;15(11):683–693. 26565774 10.1089/vbz.2015.1810 PMC12407189

[16] Baker M, Hughes HR, Naqvi SH, et al. Reassortant Cache Valley virus associated with acute febrile, non-neurologic illness, Missouri. Clin Infect Dis. 2021 Feb 25;ciab175. 10.1093/cid/ciab175 PMC12416920 33630998

[17] Nguyen NL, Zhao G, Hull R, et al. Cache valley virus in a patient diagnosed with aseptic meningitis. J Clin Microbiol. 2013 Jun;51(6):1966–1969. 23515536 10.1128/JCM.00252-13 PMC3716113

[18] Sexton DJ, Rollin PE, Breitschwerdt EB, et al. Life-Threatening Cache Valley virus infection. N Engl J Med. 1997;336(8):547–549. 9023091 10.1056/NEJM199702203360804

[19] Campbell GL, Mataczynski JD, Reisdorf ES, et al. Second human case of Cache Valley virus disease. Emerging Infect Dis.. 2006;12(5):854–856. 10.3201/eid1205.051625 PMC3374447 16704854

[20] Andreadis TG, Armstrong PM, Anderson JF, et al. Spatial-temporal analysis of Cache Valley virus (Bunyaviridae: Orthobunyavirus) infection in anopheline and culicine mosquitoes (diptera: culicidae) in the Northeastern United States, 1997–2012. Vector Borne Zoonotic Dis. 2014 Oct;14(10):763–773. 25325321 10.1089/vbz.2014.1669 PMC4208611

[21] Buescher EL, Byrne RJ, Clarke GC, et al. Cache Valley virus in the Del Mar Va Peninsula. I. Virologic and serologic evidence of infection. Am J Trop Med Hyg. 1970 May;19(3):493–502. 4392807 10.4269/ajtmh.1970.19.493

[22] Work TH. Serological evidence of Arbovirus infection in the seminole Indians of Southern Florida. Science. 1964 Jul 17;145(3629):270–272. 14171866 10.1126/science.145.3629.270

[23] Calisher CH, Sever JL. Are North American Bunyamwera serogroup viruses etiologic agents of human congenital defects of the central nervous system? Emerg Infect Dis. 1995 Oct-Dec;1(4):147–151. 8903187 10.3201/eid0104.950409 PMC2626893

[24] Wilson MR, Suan D, Duggins A, et al. A novel cause of chronic viral meningoencephalitis: Cache Valley virus. Ann Neurol. 2017 Jul;82(1):105–114. 28628941 10.1002/ana.24982 PMC5546801

[25] Chung S, Livingston CW, Edwards JF, et al. Evidence that Cache Valley virus induces congenital malformations in sheep. Vet Microbiol. 1990 Feb;21(4):297–307. 2107620 10.1016/0378-1135(90)90001-c

[26] Edwards JF, Livingston CW, Chung SI, et al. Ovine arthrogryposis and central nervous system malformations associated with in utero Cache Valley virus infection: spontaneous disease. Vet Pathol. 1989 Jan;26(1):33–39. 2492399 10.1177/030098588902600106

[27] Hayles LB, Lversen JO. Cache Valley virus: experimental infection inCuliseta inornata. Can J Microbiol. 1980 Mar;26(3):287–290. 6105910

[28] Yang F, Chan K, Marek PE, et al. Cache Valley virus inAedes japonicus japonicusMosquitoes, Appalachian region, United States. Emerg Infect Dis. 2018 Mar;24(3):553–557. 29460762 10.3201/eid2403.161275 PMC5823325

[29] Ayers VB, Huang Y-S, Lyons AC, et al. Infection and transmission of Cache Valley virus by Aedes albopictus and Aedes aegypti mosquitoes. Parasit Vectors. 2019 Jul 31;12(1):384. 31366369 10.1186/s13071-019-3643-0 PMC6670168

[30] Shan C, Xie X, Shi P-Y. Reverse genetics of zika virus. Methods Mol Biol. 2017;1602:47–58. 28508213 10.1007/978-1-4939-6964-7_4

[31] Auguste AJ, Kaelber JT, Fokam EB, et al. A newly isolated Reovirus has the simplest genomic and structural organization of any Reovirus. J Virol. 2015 Jan;89(1):676–687. 25355879 10.1128/JVI.02264-14 PMC4301156

[32] Chung SI, Livingston CW, Jr, Edwards JF, et al. Congenital malformations in sheep resulting from in utero inoculation of Cache Valley virus. Am J Vet Res. 1990 Oct;51(10):1645–1648. 2122779

[33] Wilson SN, Lopez K, Coutermash-Ott S, et al. La Crosse virus shows strain-specific differences in pathogenesis. Pathogens. 2021 Mar 29;10(4): 400. 33805389 10.3390/pathogens10040400 PMC8066585

[34] Crandell RA, Livingston CW, Jr, Shelton MJ. Laboratory investigation of a naturally occurring outbreak of arthrogryposis-hydranencephaly in Texas sheep. J Vet Diagn Invest. 1989 Jan;1(1):62–65. 2488650 10.1177/104063878900100117

[35] McConnell S, Livingston C, Jr, Calisher CH, et al. Isolations of Cache Valley virus in Texas, 1981. Vet Microbiol. 1987 Jan;13(1):11–18. 3101276 10.1016/0378-1135(87)90093-9

[36] Marin-Lopez A, Calvo-Pinilla E, Moreno S, et al. Modeling arboviral infection in mice lacking the interferon alpha/beta receptor. Viruses. 2019 Jan 8;11(1):35. 10.3390/v11010035 PMC6356211 30625992

[37] Sodergard J, Hakamies-Blomqvist L, Sainio K, et al. Arthrogryposis multiplex congenita: perinatal and electromyographic findings, disability, and psychosocial outcome. J Pediatr Orthop B. 1997 Jul;6(3):167–171. 9260644

[38] Hall JG. Arthrogryposis multiplex congenita: etiology, genetics, classification, diagnostic approach, and general aspects. J Pediatr Orthop B. 1997 Jul;6(3):159–166. 9260643

[39] Habjan M, Pichlmair A, Elliott RM, et al. NSs protein of rift valley fever virus induces the specific degradation of the double-stranded RNA-dependent protein kinase. J Virol. 2009 May;83(9):4365–4375. 19211744 10.1128/JVI.02148-08 PMC2668506

[40] Reynolds ES, Hart CE, Hermance ME, et al. An overview of animal models for arthropod-borne viruses. Comp Med. 2017 Jun 1;67(3):232–241. 28662752 PMC5482515

