# Supplementary material for: Novel murine models for studying Cache Valley virus pathogenesis and in utero transmission
Source: Emerg Microbes Infect. 2021 Aug 18;10(1):1649–59. doi: 10.1080/22221751.2021.1965497 (PMC8381923; doi:10.1080/22221751.2021.1965497)
Supplement: Supplemental Material [file TEMI_A_1965497_SM1832.docx]

**Supplementary Materials**

**
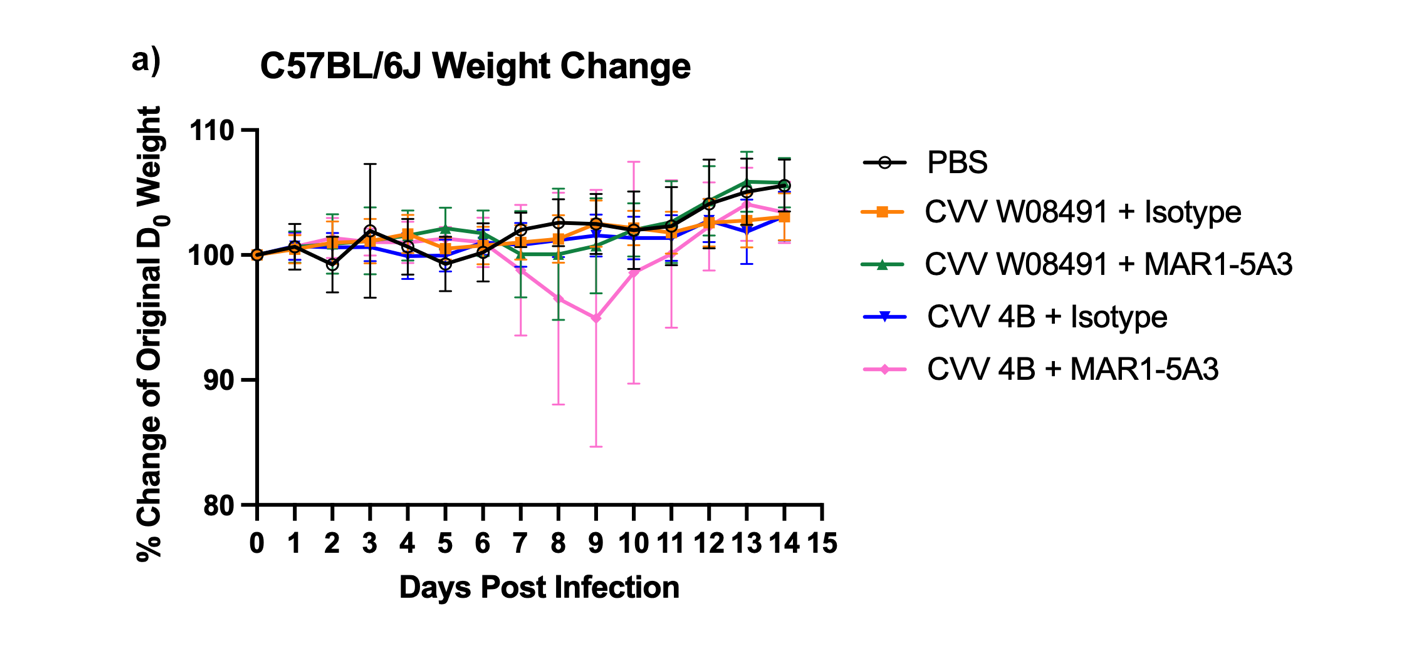
**

**Figure S1**. **CVV does not present with severe disease in C57BL/6J mice.** Seven-week-old mice (*n*=8/group) were administered 250µg of either MAR1-5A3, or Isotype IgG_1_ Control one day prior to inoculation, and the following day inoculated subcutaneously with 10^4^ plaque forming units (PFU) of virus. Following infection, mice were given two additional 100µg doses of MAR1-5A3, Isotype IgG_1_ and PBS, on 1- and 4-days post infection (DPI). Weight change was measured daily for 14 DPI. No viremia was detected in these mice throughout the study period. Each data point plotted represents the mean values and error bars indicate standard deviation. The limit of detection (LOD) is indicated with a dotted line. Statistical significance among groups was analyzed by a mixed effects analysis with a Dunnett’s multiple comparison test. Statistically significant values are denoted by * (p<0.05).

**Table S1. Meta-data for viruses used in this study.**

| **Strain Name** | **Source** | **Location of Isolation** | **Year of Isolation** | **Accession Number** | **Cell Type** | **Stock Titer (PFU/mL)** | **Viral Lineage** |
| --- | --- | --- | --- | --- | --- | --- | --- |
| 4B  MZ612419, MZ612420 and MZ612423 | *Ae. japonicus* | Blacksburg, VA | 2015 | Pending | Vero | 7.70 X 10^7^ | 2 |
|  |  |  |  |  | C6/36 | 1.33 X 10^5^ |  |
| W08491  MZ612418, MZ612421 and MZ612422 | Cx. tarsalis | North Dakota | 2005 | Pending | Vero | 1.09 X 10^7^ | 1 |
|  |  |  |  |  | C6/36 | 5.72 X 10^3^ |  |

**Table S2.** Cache valley virus infected IFN-αβR^-/-^ mice demonstrated significant differences in hemogram measurements during infection.

| **Measurements** | **Day 1** | | | **Day 3** | | | **Day 5** | | |
| --- | --- | --- | --- | --- | --- | --- | --- | --- | --- |
|  | **Negative Control** | **CVV W08491** | **CVV 4B** | **Negative Control** | **CVV W08491** | **CVV 4B** | **Negative Control** | **CVV W08491** | **CVV 4B** |
| **RBC** | 9.316 ± 0.353 | 9.63 ± 0.184 | 9.353 ± 0.606 | 9.346 ± 0.503 | 9.726 ± 0.355 | 10.022 ± 0.349 | 9.530 ± 0.409 | 9.772 ± 0664 | 9.760 ± 0.372 |
| **Hemoglobin** | 15.700 ± 0.430 | 15.720 ± 0.259 | 15.775 ± 0.222 | 14.840 ± 0.971 | 15.520 ± 0.536 | 15.640 ± 0.422 | 15.500 ± 0.200 | 15.520 ± 0.455 | 15.500 ± 0.735 |
| **Hematocrit** | 49.080 ± 1.285 | 50.080 ± 0.968 | 48.725 ± 2.516 | 49.160 ± 1.385 | 50.060 ± 1.885 | 51.320 ± 1.782 | 51.180 ± 1.385 | 50.820 ± 2.752 | 50.750 ± 1.658 |
| **MCV** | 52.700 ± 0.735 | 52.020 ± 0.867 | 52.125 ± 1.024 | 52.620 ± 1.026 | 51.460 ± 0.207 | 51.220 ± 0.522 | 52.600 ± 0.418 | 52.600 ± 0.418 | 52.025 ± 0.574 |
| **MCHC** | 32.000 ± 0.510 | 31.400 ± 0.752 | 32.425 ± 1.253 | 30.180 ± 1.441 | 31.000 ± 0.640 | 30.520 ± 1.516 | 30.960 ± 1.539 | 30.620 ± 1.810 | 30.525 ± 0.645 |
| **NRBC Sysmex** | 13.600 ± 4.529 | 7.460 ± 1.379 | 16.325 ± 10.106 | 7.820 ± 3.273 | 9.300 ± 2.874 | 6.660 ± 4.442 | 9.380 ± 4.307 | 14.200 ± 8.997 | 6.000 ± 1.903 |
| **RDW-CV** | 15.800 ± 0.400 | 15.980 ± 0.455 | 15.500 ± 0.913 | 16.300 ± 0.200 | 16.540 ± 0.167 | 16.500 ± 0.167 | 16.380 ± 0.342 | 16.880 ± 0.492 | 16.100 ± 0.572 |
| **Retic %** | 4.080 ± 1.099 | 3.660 ± 0.428 | 3.700 ± 0.294 | 4.700 ± 1.913 | 3.800 ± 0.235 | 3.760 ± 0.261 | 3.820 ± 0.277 | 3.980 ± 0.377 | 3.850 ± 0.208 |
| **Reticulocyte #** | 376.640 ± 88.277 | 351.980 ± 39.109 | 342.525 ± 16.715 | 433.380 ± 148.445 | 369.340 ± 21.304 | 375.680 ± 31.821 | 363.680 ±29.170 | 389.300 ± 55.713 | 375.825 ± 26.638 |
| **WBC** | 8.342 ± 1.595 | 8.182 ± 0.952 | 9.155 ± 1.975 | 5.434 ± 1.506 | 4.298 ± 1.334 | 7.178 ± 1.512 | 9.592 ± 2.956 | 7.234 ± 1.137 | 5.125 ± 0.974* |
| **Neutrophils** | 1.460 ± 0.699 | 0.900 ± 0.212 | 1.250 ± 0.332 | 0.780 ± 0.249 | 0.720 ± 0.130 | 1.060 ± 0.241 | 2.280 ± 1.221 | 3.040 ± 1.260 | 1.875 ± 0.330 |
| **Lymphocytes** | 5.960 ± 1.498 | 6.580 ± 1.038 | 6.975 ± 2.069 | 4.140 ± 1.390 | 3.220 ± 1.281 | 5.480 ± 1.305 | 6.060 ± 2.115 | 3.020 ± 1.375 | 2.100 ± 0.658* |
| **Monocytes** | 0.620 ± 0.228 | 0.400 ± 0.071 | 0.550 ± 0.058 | 0.440 ±0.182 | 0.280 ± 0.084 | 0.500 ± 0.394 | 0.940 ± 0.550 | 0.900 ± 0.394 | 1.000 ± 0.216 |
| **Eosinophils** | 0.220 ± 0.045 | 0.220 ± 0.045 | 0.300 ± 0.000* | 0.040 ± 0.055 | 0.060 ± 0.089 | 0.120 ± 0.045 | 0.240 ± 0.114 | 0.180 ± 0.084 | 0.100 ± 0.000 |
| **Basophils** | 0.080 ± 0.045 | 0.100 ± 0.000 | 0.100 ± 0.000 | 0.020 ± 0.045 | 0.000 ± 0.000 | 0.020 ± 0.045 | 0.060 ± 0.055 | 0.080 ± 0.045 | 0.075 ± 0.050 |
| **Platelets** | 1142.800 ± 281.557 | 1287.600 ± 52.662 | 1308.500 ± 28.219 | 1314.400 ± 239.755 | 1428.400 ± 104.820 | 1096.600 ± 672.587 | 1473.200 ± 148.571 | 1175.400 ± 305.062 | 916.750 ± 202.253** |
| **MPV** | 7.080 ± 0.526 | 6.600 ± 0.071 | 6.775 ± 0.275 | 6.800 ± 0.141 | 7.000 ± 0.141 | 7.060 ± 0.709 | 7.060 ± 0.329 | 7.120 ± 0.356 | 7.000 ± 0.383 |

Each data point represents the mean and standard deviations. Statistically significant values are denoted by * (p<0.05).

*Red Blood Cells (RBC), Hemoglobin, Hematocrit, Mean corpuscular volume (MCV), Mean corpuscular hemoglobin volume (MCHC), Nucleated Red Blood Cell Sysmex (NRBC Sysmex), Red blood cell distribution width (RDW-CV), Reticulocytes percent (Retic %), Reticulocyte number, White Blood Cells (WBC), Neutrophil, Lymphocytes, Monocytes, Eosinophils*

**Table S3.** Cache Valley virus infected IFN-αβR^-/-^ mice demonstrated significant histopathology across tissues during infection.

Each data point was graded on a scale from 0-3 where 0=no lesions observed, 1=mild lesions observed, 2=moderate lesions observed, and 3=severe lesions observed. Individual scores for each parameter were then summed for each tissue for a total histopathology score for each organ.

**Table S4.** Cache valley virus infected IFN-αβR^-/-^ mice demonstrated significant differences in cytokine and chemokine responses during infection.

| **Parameters** | **Day 1** | | | **Day 3** | | | **Day 5** | | |
| --- | --- | --- | --- | --- | --- | --- | --- | --- | --- |
|  | **PBS** | **CVV W08491** | **CVV 4B** | **PBS** | **CVV W08491** | **CVV 4B** | **PBS** | **CVV W08491** | **CVV 4B** |
| CXCL1 | 22.591 ± 8.013 | 18.341 ± 4.198 | 20.808 ± 2.715 | 17.811 ± 3.535 | 75.685 ± 71.821 | 86.150 ± 73.960 | 16.2525 ± 3.773 | 154.581 ± 170.972 | 190.609 ± 160.415* |
| GM-CSF | 0.605 ± 0.641 | 0.200 ± 0.035 | 0.347 ± 0.222 | 0.312 ± 0.136 | 0.303 ± 0.088 | 0.225 ± 0.040 | 0.230 ± 0.049 | 0.298 ± 0.107 | 0.377 ± 0.098* |
| IFN-𝛾 | 0.689 ± 0.204 | 0.981 ± 0.671 | 0.430 ± 0.182* | 2.720 ± 3.659 | 25.556 ± 19.620* | 9.738 ± 11.521 | 0.755 ± 0.737 | 268.245 ± 305.249 | 269.594 ± 322.283 |
| IL-1β | 0.696 ± 0.430 | 0.299 ± 0.023 | 0.384 ± 0.128 | 0.354 ± 0.124 | 1.056 ± 1.117 | 0.383 ± 0.139 | 0.301 ± 0.054 | 0.514 ± 0.273 | 0.653 ± 0.294* |
| IL-10 | 5.160 ± 1.811 | 5.731 ± 1.878 | 4.798 ± 0.837 | 7.093 ± 1.819 | 13.255 ± 3.798* | 7.506 ± 1.146* | 4.343 ± 0.629 | 11.556 ± 5.797* | 7.873 ± 5.299 |
| IL-12p70 | 0.886 ± 0.531 | 1.338 ± 1.402 | 0.815 ± 0.443 | 0.815 ± 0.364 | 0.873 ± 0.537 | 0.645 ± 0.146 | 0.644 ± 0.194 | 3.191 ± 3.253 | 4.523 ± 4.094 |
| IL-13 | 0.713 ± 0.549 | 0.308 ± 0.049 | 0.463 ± 0.244 | 0.515 ± 0.103 | 0.714 ± 0.233 | 0.618 ± 0.154 | 0.341 ± 0.083 | 0.796 ± 0.807 | 0.931 ± 0.653 |
| IL-17A | 1.318 ± 0.440 | 0.830 ± 0.323* | 0.788 ± 0.322 | 0.485 ± 0.207 | 1.523 ± 0.510* | 0.783 ± 0.278* | 1.180 ± 0.839 | 0.929 ± 0.375 | 0.991 ± 0.533 |
| IL-2 | 4.394 ± 5.174 | 1.556 ± 0.732 | 3.017 ± 2.459 | 2.426 ± 1.329 | 2.466 ± 1.283 | 2.256 ± 0.542 | 1.951 ± 0.608 | 1.736 ± 0.790 | 3.511 ± 2.011 |
| IL-4 | 1.838 ± 1.905 | 0.423 ± 0.069 | 1.038 ± 0.615 | 0.709 ± 0.443 | 0.680 ± 0.292 | 0.530 ± 0.099 | 0.523 ± 0.155 | 0.506 ± 0.179* | 1.077 ± 0.317* |
| IL-5 | 4.601 ± 1.879 | 2.309 ± 0.385* | 3.404 ± 0.665* | 6.395 ± 1.826 | 38.674 ± 30.874* | 57.238 ± 64.938 | 2.869 ± 0.598 | 72.351 ± 112.187 | 36.086 ± 55.504 |
| IL-6 | 12.631 ± 9.597 | 6.000 ± 1.227 | 17.583 ± 2.641* | 13.017 ± 3.871 | 19.536 ± 15.370 | 16.414 ± 7.413 | 7.920 ± 2.733 | 32.420 ± 44.725 | 60.384 ± 51.114 |
| IL-9 | 0.578 ± 0.247 | 0.225 ± 0.062* | 0.468 ± 0.295 | 0.443 ± 0.193 | 4.880 ± 7.537 | 2.741 ± 2.179* | 0.264 ± 0.069 | 5.908 ± 8.053 | 4.314 ± 6.227 |
| MCP1 | 64.399 ± 11.538 | 60.809 ± 16.146 | 60.158 ± 17.366 | 105.631 ± 40.350 | 795.294 ± 583.575* | 503.138 ± 338.957* | 51.460 ± 11.365 | 716.211 ± 536.874* | 1003.764 ± 769.977* |
| MIP1⍺ | 1.516 ± 0.761 | 1.058 ± 0.128 | 1.409 ± 0.418 | 1.050 ± 0.140 | 1.843 ± 1.348 | 1.215 ± 0.299 | 1.260 ± 0.216 | 2.645 ± 2.647 | 7.410 ± 5.870 * |
| MIP1β | 13.271 ± 1.121 | 11.505 ± 2.406 | 12.013 ± 3.559 | 11.958 ± 2.031 | 27.606 ± 19.550 | 17.200 ± 5.564 | 14.981 ± 0.908 | 55.024 ± 53.613 | 124.403 ± 94.232 * |
| TNF-⍺ | 3.488 ± 1.461 | 2.613 ± 0.481 | 2.928 ± 0.500 | 5.446 ± 3.625 | 24.823 ± 27.422 | 9.355 ± 4.844 | 3.478 ± 0.662 | 40.976 ± 47.749 | 80.643 ± 88.224 |

Each data point represents the mean and standard deviations. Statistically significant values are denoted by * (p<0.05).

*(CXCL1) Chemokine C-X-C motif ligand 1, (GM-CSF) Granulocyte-macrophage colony-stimulating factor, (IFN-𝛾) Interferon gamma, (IL-1β) Interleukin 1 beta, (IL-10) Interleukin 10, (IL-12p70) Interleukin 12, (IL-13) Interleukin 13, (IL-17A) Interleukin 17A, (IL-2) Interleukin 2, (IL-4) Interleukin 4, (IL-5) Interleukin 5, (IL-6) Interleukin 6, (IL-9) Interleukin 9, (MCP1) Monocyte chemoattractant protein 1, (MIP1⍺) Macrophage Inflammatory Protein-1 alpha, (MIP1β) Macrophage Inflammatory Protein-1 beta, (TNF-⍺) Tumor necrosis factor-alpha*
